# SR-FEINR: Continuous Remote Sensing Image Super-Resolution Using Feature-Enhanced Implicit Neural Representation

**DOI:** 10.3390/s23073573

**Published:** 2023-03-29

**Authors:** Jinming Luo, Lei Han, Xianjie Gao, Xiuping Liu, Weiming Wang

**Affiliations:** 1 School of Mathematics and Science, Dalian University of Technology, Dalian 116024, China; 2 Department of Basic Sciences, Shanxi Agricultural University, Jinzhong 030801, China

**Keywords:** remote sensing image super-resolution, implicit neural representation, position encoding

## Abstract

Remote sensing images often have limited resolution, which can hinder their effectiveness in various applications. Super-resolution techniques can enhance the resolution of remote sensing images, and arbitrary resolution super-resolution techniques provide additional flexibility in choosing appropriate image resolutions for different tasks. However, for subsequent processing, such as detection and classification, the resolution of the input image may vary greatly for different methods. In this paper, we propose a method for continuous remote sensing image super-resolution using feature-enhanced implicit neural representation (SR-FEINR). Continuous remote sensing image super-resolution means users can scale a low-resolution image into an image with arbitrary resolution. Our algorithm is composed of three main components: a low-resolution image feature extraction module, a positional encoding module, and a feature-enhanced multi-layer perceptron module. We are the first to apply implicit neural representation in a continuous remote sensing image super-resolution task. Through extensive experiments on two popular remote sensing image datasets, we have shown that our SR-FEINR outperforms the state-of-the-art algorithms in terms of accuracy. Our algorithm showed an average improvement of 0.05 dB over the existing method on ×30 across three datasets.

## 1. Introduction

With the development of satellite image processing technology, the application of remote sensing has increased [1,2,3,4,5]. However, low spatial, spectral, radiometric, and temporal resolutions of current image sensors and complicated atmospheric conditions make it hard to use remote sensing. Consequently, extensive super-resolution (SR) methods have been proposed to improve the low quality and low resolution of remote sensing images.

SR reconstruction is a method used for generating high-resolution remote sensing images, which combines a large number of images with similar content. Generally, remote sensing image SR reconstruction algorithms can be classified into three categories: single remote sensing image SR reconstruction [6,7,8,9,10,11], multiple remote sensing image SR reconstruction [12,13], and multi/hyperspectral remote sensing image SR reconstruction [14]. Since the latter two approaches have poor SR effects, registration fusion, multi-source information fusion, and other issues, more research studies have been focusing on single remote sensing image SR reconstruction.

Single remote sensing image SR (SISR) methods can be divided into two categories based on the generative adversarial network and the convolution neural network. Although both GAN-based networks and CNN-based networks can achieve good results in SISR, they can only scale the low-resolution (LR) image with an integer factor, which makes the obtained high-resolution (HR) image inconvenient for downstream tasks. One way to solve this problem is to represent a discrete image continuously with implicit neural representation. Continuous image representation allows recovering arbitrary resolution imaging by modeling the image as a function defined in a continuous domain. For a continuous domain, the best way to describe an image is to fit this image as a function of continuous coordinates. Our method is motivated by recent advances in implicit neural representation for 3D shape reconstruction [15]. The concept behind implicit functions is to represent a signal as a function that maps coordinates to the corresponding signal (e.g., signed distance to a 3D object surface). In remote sensing image super-resolution, the signals can be the RGB values of an image. Multi-layer perceptron (MLP) is a common way to implement implicit neural representation. Instead of fitting unique implicit functions for each object, encoder-based approaches are suggested to predict a latent code for each item in order to share information across instances. The implicit function is then shared by all objects, and it accepts the latent code as an extra input. Although the encoder-based implicit function method is effective in a 3D challenge, it can only successfully represent simple images and is unable to accurately represent remote sensing images.

To solve the problem of the expression ability of encoder-based implicit neural representations, this paper explores different positional encoding methods in image representation for the image SR task, and proposes a novel feature-enhanced MLP network to enhance the approximation ability of the original MLP. Our main contributions are as follows:

1. We are the first to adopt the implicit neural representation into remote sensing image SR tasks. With our method, one can obtain significant improvements in AID and UC Merced datasets.
2. We propose a novel feature-enhanced MLP architecture to make use of the feature information of the low-resolution image.
3. The performances of different positional encoding methods are investigated in implicit neural representations for continuous remote sensing image SR tasks.

## 2. Related Works

In this section, we will briefly review the implicit neural representation and the related methods, including positional encoding and continuous image SR.

### 2.1. Implicit Neural Representation

The implicit neural representation is essentially a continuously differentiable function that maps the coordinates into the signals. It has been widely used in many fields, such as shape parts [16,17], objects [18,19,20,21], or scenes [22,23,24,25]. The implicit neural representation is a data-driven method. It is trained from some form of data as a signal distance function. Many 3D-aware image generation methods use convolutional architectures. Park et al. [18] proposed using neural networks to fit scalar functions for the representation of 3D scenes. Mildenhall et al. [26] proposed a neural radiance field (Nerf) to implicitly represent a scene. It takes images of the same scene taken from different viewpoints as inputs and uses a neural network to learn a static 3D scene implicitly. Based on these images, the trained neural network can render images from any perspective. However, the present work based on implicit neural representation does not perform very well in the spatial and temporal derivatives. In terms of image generation, Chen et al. [27] proposed a local implicit image function (LIIF). It feeds the coordinates and the features corresponding to the MLP and outputs a RGB signal for the coordinates. Since the coordinates of images with arbitrary resolution are continuous, LIIF can represent images with arbitrary resolutions.

### 2.2. Positional Encoding

In order to capture the positional relationships, a method called positional encoding is introduced in [28,29]. Positional encoding is essentially a map from a position space to a high-dimensional vector space. For the continuous image SR task, 2D image coordinates are mapped into high-dimensional vectors. The common method used in [29] employs sinusoidal positional encoding by manually designing. The performance of the hand-designed approach depends on the weights of the sinusoidal positional encoding, which lacks flexibility. In order to improve the flexibility of the positional encoding, Parmar et al. [30] introduced a learnable embedding vector for each position for 1D cases. Although the trainable embedding method has the potential to capture more complex positional relationships, the learnable parameters are largely increased with the increasing dimensionality of the positional input coordinates. For the purpose of capturing more complex position relationships, for instance, the similarity of positions in an image, a novel learnable positional encoding was proposed in [31]. In their proposed method, a function is learned to map multi-dimensional positions into a vector space based on the Fourier transform. The obtained vectors are fed into the MLP. In our work, we will also focus on the learnable positional encoding method.

### 2.3. Continuous Image SR

Image SR is a reconstruction task that restores a realistic and more detailed high-resolution image from a LR image. It is an important class of computer vision image processing techniques. However, it is an ill-posed problem because a specific LR image corresponds to a set of possible high-resolution images. Due to the powerful characterization and extraction capabilities of deep learning in both low-resolution and high-resolution spaces, deep learning-based image SR tasks have significantly improved in both qualitative and quantitative terms. Dong et al. [32] were the first to research single natural image SR based on deep learning, called SRCNN. It uses a bicubic interpolation to scale a LR image to a target size. Then, these images are fed into a three-layer convolutional network to fit a nonlinear map. The output is a HR image. In [33], a novel network, FSRCNN, was proposed to improve the inference speed of SRCNN. However, the SRCNN model not only learns how to generate high-frequency information, but it also needs to reconstruct low-frequency information, which greatly reduces its efficiency. Kim et al. [34] proposed VDSR to increase the depth of the network by employing the residual connect. Remote sensing images are different from natural images, as they often have coupled objects and environments, and the images span a wide range of scales. In order to make full use of the environmental information, Lei et al. [35] proposed a VDSR-based network called a local–global combined network (LGCNet).

It is evident that all the methods mentioned above upsample the input LR images before feeding them into the model for learning, which slows down the convergence speed of the model and also greatly increases the memory overhead. The ESPCN model [36] proposed a sub-pixel convolution operation as an efficient, fast, and non-parametric pixel rearrangement upsampling method, which significantly improved the training efficiency of the network. To further improve the expressive power of the model, the SRResNet model was proposed in [37], which utilized the residual module widely used in image classification tasks. At the same time, the confrontational generation loss function was first adopted to the image SR problem, which achieved satisfactory results. In [38], the EDSR model was proposed to further optimize the above network structure. Additionally, the performance of the EDSR model was further improved by removing the batch normalization layer and the second activation layer from the residual module. Later, several models were proposed to enhance the network’s performance, including the RDN model [39] and the RCAN model [40]. To adaptively fuse the extracted multi-scale information, Wang et al. [41] proposed an adaptive multiscale feature fusion network for SR of remote sensing images.

**Figure 1:**
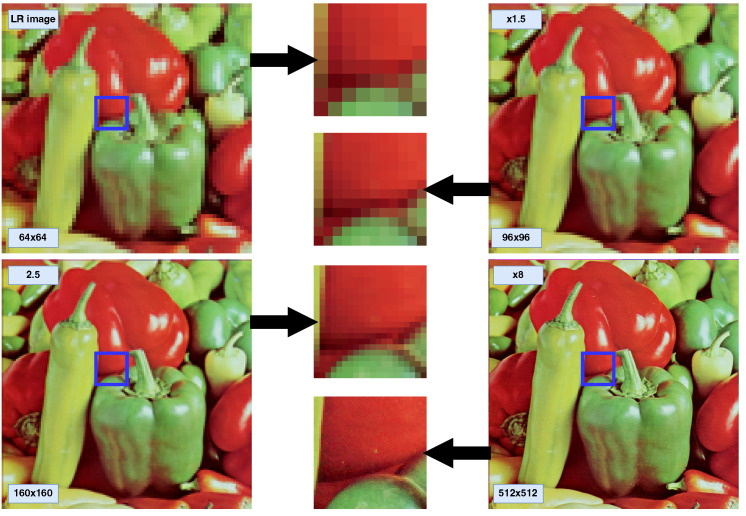
An image in the continuous domain can be presented in arbitrary high resolution.

However, the above methods can only upsample an image to a specific scale. To generate the HR image of arbitrary resolution, MetaSR, ref. [42] introduced a meta-upscale module, which employs a single model to upsample the input image to arbitrary resolution by dynamically predicting weights. However, it cannot achieve satisfactory results for the resolutions outside of the training distribution. Therefore, Chen et al. [27] proposed a local implicit image function (LIIF) by taking advantage of the neural implicit representation. In their method, the coordinates and the features corresponding are fed to the MLP to obtain a RGB signal. Since the coordinates are continuous, the HR image can be presented in arbitrary resolution. However, LIIF ignores the influence of positional encoding on image generation. Therefore, in this work, the coordinate was encoded to obtain more high-dimensional information about the coordinates, which can produce more realistic HR images. Figure 1 shows the results of our method, which can scale the input image into an arbitrary resolution.

## 3. Method

Image SR is a common task in computer vision that outputs a high-resolution image $I_{H}$ based on the input LR image $I_{L}$. In other words, for each continuous coordinate p in the high-resolution image $I_{H}$, we need to calculate a signal at this coordinate, denoted as $c_{p}$. In the image SR task, the signal for a coordinate is the RGB value. In the following section, we will introduce the details of our method.

### 3.1. Network Overview

**Figure 2:**
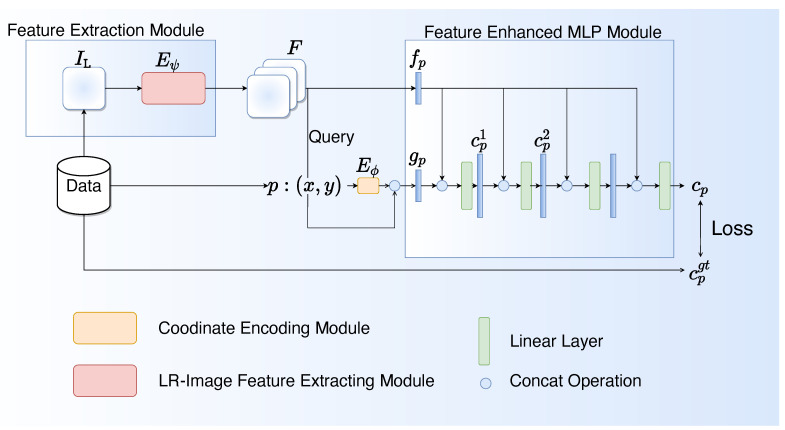
The architecture of the proposed model. The blue rectangles indicate the feature vectors corresponding to the coordinates.

The main part of the proposed network is illustrated in Figure 2. It is composed of three major components: the feature extraction module ($E_{\psi }$), the positional encoding module ($E_{\phi }$), and the feature-enhanced MLP module ($M_{\theta }$).

For a given discrete image $I\in R^{H\times W\times 3}$, we define the coordinate bank $B_{I}$ as a subset of ${\left[-1,1\right]}^{2}$:

(1) $$\begin{array}{ccc} B_{I} & = & \{\left(x,y\right)|x\in \left\{-1+\frac{1}{H},-1+\frac{3}{H},\cdots ,1-\frac{1}{H}\right\},\\ & & y\in \{-1+\frac{1}{W},-1+\frac{3}{W},\cdots ,1-\frac{1}{W}\}\} \end{array}$$

For a LR image $I_{L}$, the feature extraction module $E_{\psi }$ is used to extract the features $F\in R^{(\#B_{I_{L}})\times l}$ of the LR image. For a coordinate $p\in B_{I_{H}}$ in a HR image $I_{H}$, the feature at p can be set as the nearest point feature in $B_{I_{L}}$, which can be formulated as:

(2) $$f_{p}=F_{q^{*}},q^{*}=\underset{q\in B_{I_{L}}}{\arg\min}d\left(p,q\right).$$

The positional encoding module $E_{\phi }$ is used to encode the coordinate p into a high-dimensional space. The output encoding vector at this position is formulated as:

(3) $$g_{p}=\mathrm{concat}\left(E_{\phi }\left(p\right),p\right).$$

We will discuss the performances of three commonly used positional encoding methods in Section 5.2.

With the feature $f_{p}$ and the encoding vector $g_{p}$, the feature-enhanced MLP module $M_{\theta }$ is used to reconstruct the signal $c_{p}$, which can be formulated as:

(4) $$c_{p}=M_{\theta }\left(f_{p},g_{p}\right).$$

Consequently, for any coordinate p∈P, P is the set of coordinates p in the high-resolution image $I_{H}$, and the L1 loss is used as the reconstruction loss:

(5) $$L=\sum_{p\in P}\left|\right|c_{p}-c_{p}^{gt}{\left|\right|}_{1}^{2},$$

The complete training and inference processes are presented in Algorithm 1 and Algorithm 2, respectively.
**Algorithm 1:** Training process of continuous super-resolution using SR-FEINR.
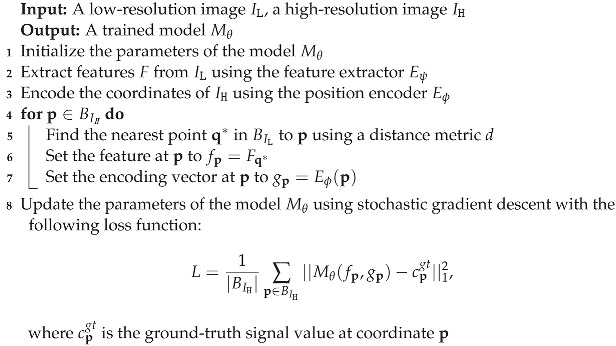


**Algorithm 2:** Inference process of continuous super-resolution using SR-FEINR

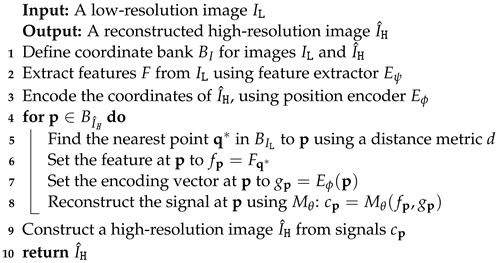



### 3.2. Feature Extraction Module and Positional Encoding Module

#### 3.2.1. Feature Extraction

As mentioned in [27], we used EDSR and RDN to extract the features of the low-resolution image. The feature extraction process in EDSR includes inputting a low-resolution image, extracting high-level features through convolutional layers, enhancing features through residual blocks, fusing features through feature fusion modules, and outputting a feature map. The feature extraction process in RDN includes inputting a low-resolution image, extracting feature maps through convolutional layers and residual dense networks, expanding features through feature expansion modules, fusing features through feature fusion modules, and finally upsampling and reconstructing the image.

For a low-resolution image $I_{L}\in R^{H\times W\times 3}$, to enrich the information of each latent code in the feature space, we update the features using the feature-unfolding method, which can be formulated as:

(6) $$F_{i}^{\prime }=\mathrm{concat}\left({\left\{F_{j}\right\}}_{d(i,j)<\epsilon }\right).$$

Afterward, we obtain the features of the low-resolution image *F*; the features of the continuous coordinate $f_{p}$ can be calculated using Equation (2) and fed into the feature-enhanced MLP module $M_{\theta }$.

#### 3.2.2. Positional Encoding

To encode the coordinate p, we use the following equation:

(7) $$\begin{array}{ccc} E(p) & = & (\sin\left(\omega_{0}\pi p\right),\cos\left(\omega_{0}\pi p\right),\sin\left(\omega_{1}\pi p\right),\\ & & \cos\left(\omega_{1}\pi p\right),\cdots \sin\left(\omega_{n}\pi p\right),\cos\left(\omega_{n}\pi p\right)), \end{array}$$

where $\omega_{0}$, $\omega_{1}$, …, and $\omega_{n}$ are coefficients and *n* is related to the dimension of the encoding space.

**Figure 3:**
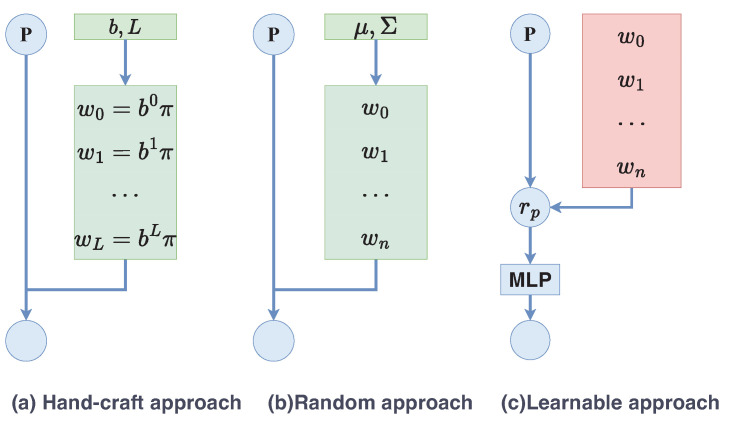
The structures of three positional encoding methods. The blue circle *P* represents the coordinate. The green rectangles indicate the hyperparameters of the Fourier features. The red rectangle indicates the learnable parameters.

As illustrated in Figure 3, the details of three common positional encoding methods are described, which are the hand-craft approach, the random approach, and the learnable approach. In the hand-craft approach, $\omega_{i}$ is fixed as $\omega_{0}=b^{0},\cdots ,\omega_{n}=b^{L}$, where *b* and *L* are hyperparameters. The difference between the random approach and the normal positional encoding is that the weights $\omega_{i}$ are randomly selected and not specified. The weights $\omega_{i}$ are sampled from a normal distribution N(μ,Σ), where μ and Σ are hyperparameters.

For the learnable approach, the encoding vector of each position is represented as a trainable code by a learnable mapping of the coordinate. A major advantage of this method for multidimensional coordinates is that it is naturally inductive and can handle test samples with arbitrary lengths. Another major advantage is that the number of parameters does not increase with the sequence length. This method is composed of two components: learnable Fourier and a MLP layer. To extract useful features, learnable Fourier features map an *M*-dimensional position p into an *F*-dimensional Fourier feature vector called $r_{p}$. The definition of learnable Fourier features is roughly the same as Equation (7),

(8) $$\begin{array}{ccc} r_{p} & = & \frac{1}{\sqrt{F}}(\sin\left(\omega_{0}\pi p\right),\cos\left(\omega_{0}\pi p\right),\sin\left(\omega_{1}\pi p\right),\\ & & \cos\left(\omega_{1}\pi p\right),\cdots \sin\left(\omega_{n}\pi p\right),\cos\left(\omega_{n}\pi p\right)), \end{array}$$

where $\omega_{0},\cdots ,\omega_{n}$ are trainable parameters, $n=\frac{F}{2}-1$ defines both the orientation and wavelength of the Fourier features. The linear projection coefficients $\omega_{0},\cdots ,\omega_{n}$ are initialized with a normal distribution $N(0,\gamma^{-2})$. The MLP layer is a simple neural network architecture for implicit neural representation with a GELU activation function:

(9) $$E_{\phi }\left(p\right)=\tau \left(r_{p},\eta \right),$$

where τ(.) is the perceptron parameterized by η.

Since the weights are learnable, the expression power of the encoding vector is more flexible. Therefore, in our work, we focus on learnable positional encoding.

### 3.3. Feature-Enhanced MLP for Reconstruction

In order to make use of the information in the LR image, we propose a feature-enhanced MLP module $M_{\theta }$ to reuse the feature of the LR image. The latent code $f_{p}$ at the coordinate p of the LR image and the encoded coordinate feature vector $g_{p}$ are fed into the first hidden layer of the MLP. This process is defined as

(10) $$c_{p}^{1}=h_{1}\left(f_{p},g_{p}\right),$$

where $h_{1}$ is the first hidden layer of the MLP, $c_{p}^{1}$ is the output vector of the first hidden layer.

Then we concatenate the image feature vector $f_{p}$ with the output feature of the previously hidden layer. At this point, Equation (10) is transformed into

(11) $$c_{p}^{2}=h_{2}\left(f_{p},c_{p}^{1}\right),$$

where $h_{2}$ is the second hidden layer of the MLP, $c_{p}^{2}$ is the output vector of the second hidden layer.

In our method, the MLP is constructed with five perceptron layers to obtain better results compared to LIIF [27]. The MLP model can be written as:

(12) $$c_{p}=h_{N-1}\left(f_{p},h_{N-2}\left(f_{p},h_{N-3}\left(f_{p},\cdots ,h_{1}\left(f_{p},g_{p}\right)\right)\right)\right),$$

where $h_{i}\left(.\right)$ is the *i*th hidden layer and $c_{p}$ is the predicted RGB value for coordinate p.

### 3.4. Implementation Details

Two feature extraction modules are considered in this work, which are EDSR and RDN. In the three positional encoding approaches, we chose the learnable positional encoding because it was more conducive to the learning of the network and it performed better in our experiment. As for the MLP setting of the feature-enhanced MLP network $M_{\theta }$, we chose a five-layer 256-*d* multilayer perceptron (MLP) with the GELU activation function.

## 4. Experiments

### 4.1. Experimental Dataset and Settings

In our experiment, we used a common dataset DIV2K [43] for the ablation study and two common remote sensing datasets: UC Merced [44] and AID [45]. In the field of remote sensing SISR, these datasets have been heavily utilized [35,46,47].

- AID dataset [45]: This dataset contains 30 classes of remote sensing scenes, such as an airport, railway station, square, and so on. Each class contains hundreds of images with a resolution of 600×600. In our experiment, we chose two types of scenes, an airport and a railway station, to evaluate different methods. The images in each scene were split into the train set and test set with a ratio of 8:2, and then we randomly picked five images from the train set as the valid set for each scene.
- UC Merced Dataset [44]: This dataset contains 21 classes of remote sensing scenes, such as an airport, baseball diamond, beach, and so on. Each class contains 100 images with a resolution of 256×256. We split the dataset into the train set, test set, and valid set with a ratio of 4:5:1.
- DIV2K dataset [43]: This dataset contains 1000 high-resolution natural images and corresponding LR images with scales ×2, ×3, and ×4. We used 800 images as the training set and 100 images in the DIV2k validation set as the test set, which followed prior work [27].

In our training process, the low-resolution image $I_{L}$ and the coordinate-RGB pairs $O={\left\{\left(p,c_{p}\right)\right\}}_{p\in A}$ of the high-resolution image can be obtained by the following steps: (1) the high-resolution image in the training dataset is cropped into a $48r_{i}\times 48r_{i}$ patch $I_{P}$, where $r_{i}$ is sampled from a uniform distribution U(1,4); (2) $I_{P}$ is downsampled with the bicubic interpolation method to generate its LR image $I_{L}$ with a resolution of 48×48; (3) for an original $48r_{i}\times 48r_{i}$ image patch $I_{P}$, the coordinate bank is constructed $B_{I_{P}}$. For each coordinate $p\in B_{I_{P}}$, its RGB value is denoted as $c_{p}$. Then, the coordinate–RGB pair set $I_{P}$ is constructed as $O^{\mathrm{full}}={\left\{\left(p,c_{p}\right)\right\}}_{p\in B_{I_{P}}}$; 4) the 48×48 coordinate–RGB pairs $O={\left\{\left(p,c_{p}\right)\right\}}_{p\in A}$ are randomly chosen from $O^{\mathrm{full}}$ to evaluate the network.

We implemented SRCNN, VDSR, and LGCNet based on the settings given in [48]. For other experiments, we adapted the same training settings given in [27]. Specifically, we used the Adam optimizer [49] with an initial learning rate $1\times 10^{-4}$. All of the experiments were trained for 1000 epochs with a batch size of 16, and the learning rate decayed by a factor of 0.5 every 200 epochs.

### 4.2. Evaluation Metrics

To evaluate the effectiveness of the proposed method, two commonly used evaluation indicators were used in [50,51,52,53]. The most popular method for evaluating the quality of outcomes is PSNR (the peak signal-to-noise ratio). For a RGB image, the PSNR can be calculated as follows:

(13) $$PSNR=10\log_{10}\left(\frac{255^{2}\times N_{p}}{MSE}\right).$$

where $N_{p}$ is the total number of pixels in the image and MSE is the mean squared error, which can be calculated as:

$$MSE=\frac{1}{3N_{p}}\sum_{i=1}^{N_{p}}\sum_{c=1}^{3}{\left[I{\left(i\right)}_{c}-K{\left(i\right)}_{c}\right]}^{2}$$

where $I{\left(i\right)}_{c}$ and $K{\left(i\right)}_{c}$ represent the intensity values of the *i*th pixel in the original and reconstructed images in the *c*th color channel, respectively.

The structural similarity index (SSIM) can be used to measure the similarity between two RGB images. The SSIM index can be calculated as follows:

(14) $$\mathrm{SSIM}\left(I,K\right)=\frac{\left(2\mu_{I}\mu_{K}+c_{1}\right)\left(2\sigma_{IK}+c_{2}\right)}{\left(\mu_{I}^{2}+\mu_{K}^{2}+c_{1}\right)\left(\sigma_{I}^{2}+\sigma_{K}^{2}+c_{2}\right)}$$

where $\mu_{I}$, $\mu_{K}$, $\sigma_{I}$, $\sigma_{K}$, and $\sigma_{IK}$ are the mean, standard deviation, and cross-covariance of the intensity values of the original and reconstructed images in the three color channels, respectively. The constants $c_{1}$ and $c_{2}$ are small positive constants to avoid instability when the denominator is close to zero. Note that the above equations assume that the original and reconstructed RGB images have the same resolution. If the images have different resolutions, they need to be resampled before calculating PSNR and SSIM.

## 5. Results and Analysis

In this section, we compare our method with several state-of-the-art image super-resolution methods, including the bicubic interpolation, SRCNN [32], VDSR [34], LGCNet [35], EDSR [38], and two continuous image super-resolution methods, i.e., MetaSR [42] and LIIF [27]. The bicubic interpolation, SRCNN [32], VDSR [34], LGCNet [35], EDSR [38], and RDN [39] depend on the magnified scale. These methods require different models for different upsampling scales during training, i.e., they cannot use the same model for arbitrary SR scales. EDSR-MetaSR, EDSR-LIIF, and EDSR-ours use EDSR as the feature extraction module. RDN-LIIF and RDN-ours use RDN as the feature extraction module.

### 5.1. Results on the Three Datasets

#### 5.1.1. Comparison Results on the AID Dataset

**Figure 4:**
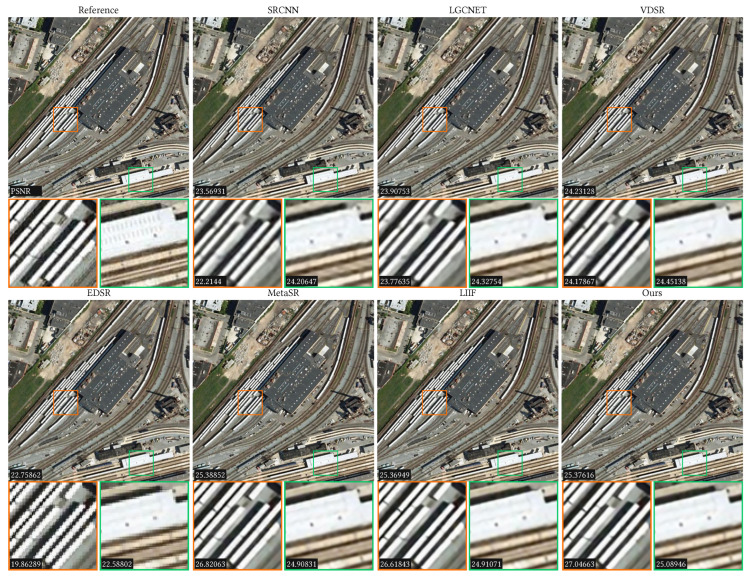
Comparison results of the ×3 scale on the railwaystation_190 scene of the AID dataset. Two local regions are zoomed in to show the detailed results. The PSNR values are listed in the bottom-left corners.

**Figure 5:**
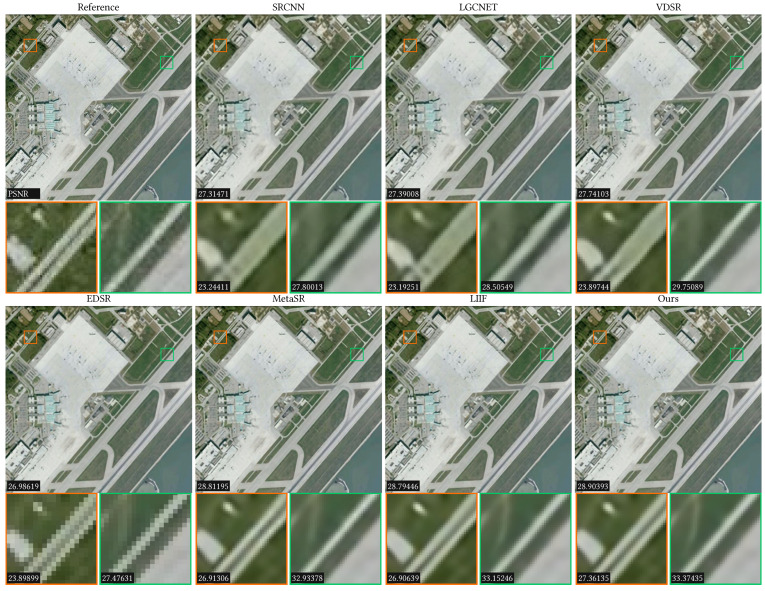
Comparison results of ×4 scale on the Airport_240 scene of the AID dataset. Two local regions are zoomed in to show the detailed results. The PSNR values are listed in the bottom-left corners.

Since the AID dataset has 30 scene categories, we only randomly selected 2 categories to show the comparison results, which are the airport and the railway station. The results are listed in Table 1 for upscale factors ×2, ×3, ×4, ×6, ×12, and ×18, where the bold text represents the best results. It can be observed that our method obtains competitive results for in-distribution scales compared to the previous methods. For out-of-distribution, our method significantly outperforms the other methods in both the PSNR and SSIM. In addition to the quantitative analysis, we also conducted qualitative comparisons, which are shown in Figure 4 and Figure 5. In Figure 4, the ×3 SR results of a railway station for different methods are shown, where two regions are zoomed in to show the details (see the red and green rectangles). The PSNR values are listed in the left-bottom corner of each image. In Figure 5, we show the ×4 SR results of an airport for different methods. From these figures, we can see that our method has the clearest details and the highest PSNR value.

#### 5.1.2. Comparison Results on UCMerced Dataset

**Figure 6:**
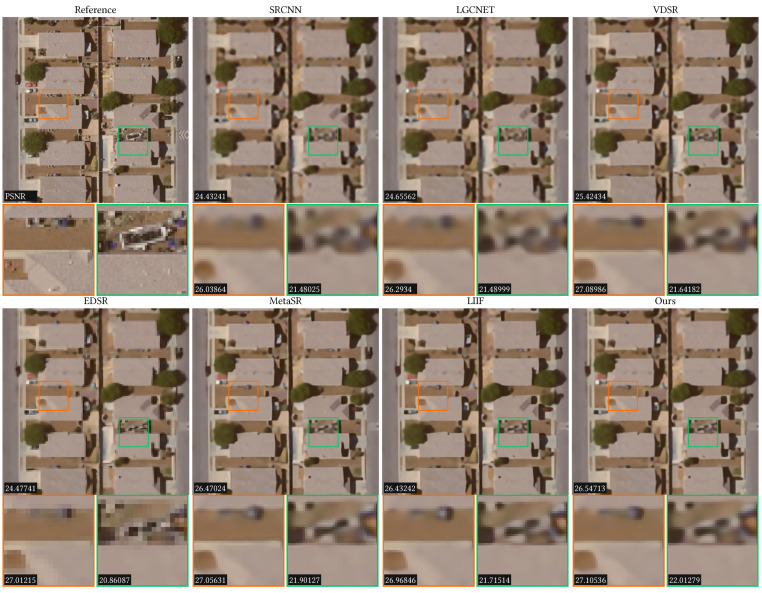
Comparison results of the ×4 scale on the dense residential_88 scene of the UC Merced dataset. Two local regions are zoomed in to show the detailed results. The PSNR values are listed in the bottom-left corners.

Different from the AID dataset, UCMerced dataset has smaller number of images and categories. Therefore, our model is trained and tested on the whole dataset. The quantitative comparison results of these methods on the UCMerced dataset are listed in Table 2. From this table we can see, our results are higher than LIIF at all magnification scales. In addition, we also visualize the SR results for different methods in Figure 6. From a visual point of view, both LIIF and our method outperform the other methods. Although the visualization results of LIIF and our method are similar, the PSNR values of the whole image and the local regions of our method are larger than LIIF, which means our method is slightly better than LIIF.

#### 5.1.3. Comparison Results on the DIV2K Dataset

Unlike the above two datasets, the images in the DIV2K dataset are mainly natural. Since our method is proposed for remote sensing image SR, we only conducted the quantitative comparisons on this dataset. In this dataset, we compare two versions of our method with Bicubic, EDSR, EDSR-MetaSR, EDSR-LIIF, and RDN-LIIF. The EDSR-ours and RDN-ours use EDSR and RDN to extract features, respectively. The comparison results are listed in Table 3. From this table, we can see that for EDSR, our method has the best performance from the ×3 scale. For the ×2 scale, LIIF and EDSR-MeatSR are better than our method as they are trained for this scale. Regarding the RDN, we only compare it with LIIF. The comparison results demonstrate that our method can achieve the best results at high scales.

### 5.2. Ablation Study

In this section, we perform ablation studies to assess the effectiveness of each module, where the EDSR is used as the feature encoder. Based on the baseline LIIF model, we progressively add the positional encoding module and feature-enhanced MLP module to evaluate their effectiveness. In order to further evaluate the effectiveness of the proposed feature-enhanced MLP module, we replace the features with coordinates and embed them into the MLP. The results of the ablation study are shown in Table 4. In this table, LIIF is our baseline. LIIF + PE is the combination of LIIF and the positional encoding module. LIIF + PE + FE is the combination of the positional encoding module and the feature-enhanced MLP module, which is our method. Based on LIIF + PE + FE, the features in the feature-enhanced MLP module are replaced with coordinates, and the resulting network is LIIF + PE + PF*. From this table, we can see that LIIF + PE + FE (our method) outperforms the LIIF at all scales except for the ×2 scale. This result proves that the learning ability of the network can be effectively improved by embedding the image features into the hidden layer of the MLP.

The positional encoding module is an important module in the proposed method. As described in Section 3.2, there are three commonly used positional encoding methods, which are the hand-craft approach, random approach, and learnable approach. Therefore, in this section, we will discuss the effectiveness of these methods on the remote sensing image SR task. The comparison results are listed in Table 5. In this table, LIIF + PE-hand represents the network with the hand-craft positional encoding method, where b=2 and L=10. i.e., $\omega_{i}=2^{i}$, i=0,1,⋯,9. LIIF + PE-random shows that the weights are chosen randomly from a normal distribution. In this network, the hyperparameters are set as μ=100 and Σ=0. The LIIF + PE-learning is the network with the learnable positional encoding method. Weights are learned through a MLP. The function τ(.) is a 2-layer MLP with the GELU activation and hidden dimensions of 256. The dimensions of the Fourier feature vector *F* are set to 768. γ is set to 10 in the normal distribution $N(0,\gamma^{-2})$. From Table 5, we can see that LIIF outperforms the other methods for in-distribution scales, which are ×2, ×3, and ×4. However, after the ×6 scale, LIIF + PE + learnable achieves the best performance among all methods. Therefore, the learnable positional encoding method is used in our network.

## 6. Conclusions

In this paper, we propose a novel network structure for continuous remote sensing image SR. By using the LIIF as our baseline, two important modules are introduced to improve its performance, which are the positional encoding module and the feature-enhanced MLP module. The positional encoding module can capture complex positional relationships by using more coordinate information. The feature-enhanced MLP module is constructed by adding prior information from the LR image to the hidden layer of MLP, which can improve the expression and learning ability of the network. Extensive experimental results demonstrate the effectiveness of the proposed method. It is worth noting that our method outperforms the state-of-the-art methods for magnifications outside of the training distribution, which is important in practical applications.

As far as we know, the inference speed of the MLP is a bit slow, which limits the application of our method. In the literature, there are some acceleration algorithms for the MLP architecture, which can be used to decrease the inference time. Therefore, we will attempt to integrate these methods into our algorithm to improve its efficiency.

## Figures and Tables

**Table 1:** Quantitative comparisons between the AID test set (PSNR (dB) and SSIM). (RS*: railway station, the bold in table is the highest value).

Dataset	Method	In-Distribution (SSIM↑/PSNR↑)			Out-of-Distribution (SSIM↑/PSNR↑)		
		×2	×3	×4	×6	×12	×18
Airport	Bicubic	0.8887/31.37	0.7949/28.39	0.7187/26.73	-	-	-
	SRCNN [32]	0.8917/31.99	0.8049/28.95	0.7336/27.22	-	-	-
	LGCNet [35]	0.8978/32.43	0.8127/29.19	0.7389/27.34	-	-	-
	VDSR [34]	0.9025/32.72	0.8211/29.56	0.7515/27.70	-	-	-
	EDSR [38]	0.9376/34.67	0.8246/29.08	0.7488/27.44	-	-	-
	EDSR-MetaSR [42]	0.9375/34.71	0.8611/30.95	0.7885/28.83	0.6822/26.47	0.5452/23.57	0.5010/22.28
	EDSR-LIIF [27]	0.9374/34.71	0.8617/30.97	0.7892/28.87	0.6849/26.54	0.5529/23.66	0.5082/22.35
	EDSR-ours	**0.9377/34.72**	**0.8617/31.00**	**0.7899/28.90**	**0.6860/26.58**	**0.5537/23.69**	**0.5091/22.39**
RS*	Bicubic	0.8863/31.70	0.7753/28.39	0.6801/26.53	-	-	-
	SRCNN [32]	0.8967/32.21	0.7992/29.03	0.7088/27.06	-	-	-
	LGCNet [35]	0.9033/32.58	0.8045/29.18	0.7111/27.11	-	-	-
	VDSR [34]	0.9088/32.88	0.8147/29.52	0.7270/27.50	-	-	-
	EDSR [38]	0.9417/35.19	0.8127/29.04	0.7217/27.30	-	-	-
	EDSR-MetaSR [42]	0.9412/35.18	0.8570/31.11	0.7690/28.76	0.6311/26.09	0.4562/22.93	0.4049/21.63
	EDSR-LIIF [27]	0.9413/35.19	0.8575/31.13	0.7696/28.78	0.6330/26.13	0.4594/22.94	0.4063/21.62
	EDSR-ours	**0.9414/35.20**	**0.8577/31.16**	**0.7711/28.83**	**0.6347/26.18**	**0.4610/23.01**	**0.4076/21.67**

**Table 2:** Mean SSIM and PSNR (dB) of the UC Merced dataset(the bold in table is the highest value).

Method	In-Distribution (SSIM↑/PSNR↑)			Out-of-Distribution (SSIM↑/PSNR↑)		
	×2	×3	×4	×6	×12	×18
Bicubic	0.8796/30.79	0.7636/27.47	0.6729/25.66	-	-	-
SRCNN [32]	0.9151/32.76	0.8095/28.83	0.7217/26.74	-	-	-
LGCNet [35]	0.9208/33.20	0.8180/29.09	0.7300/26.93	-	-	-
VDSR [34]	0.9262/33.66	0.8351/29.65	0.7486/27.43	-	-	-
EDSR [38]	0.9246/34.16	0.8158/29.86	0.6932/26.12	-	-	-
EDSR-MetaSR [42]	0.9262/34.43	0.8285/30.22	0.7454/27.91	0.6173/25.23	0.4477/22.13	0.3973/20.89
EDSR-LIIF [27]	0.9260/34.45	0.8285/30.20	0.7445/27.89	0.6185/25.23	0.4510/22.10	0.4005/20.85
EDSR-ours	**0.9259/34.46**	**0.8287/30.26**	**0.7465/27.96**	**0.6202/25.31**	**0.4521/22.20**	**0.4013/20.94**

**Table 3:** Quantitative comparison on the DIV2K validation set (PSNR (dB)), the bold in table is the highest value.

Method	In-Distribution (PSNR↑)			Out-of-Distribution (PSNR↑)				
	×2	×3	×4	×6	×12	×18	×24	×30
Bicubic	31.01	28.22	26.66	24.82	22.27	21.00	20.19	19.59
EDSR [38]	34.55	30.90	28.94	-	-	-	-	-
EDSR-MetaSR [42]	34.64	30.93	28.92	26.61	23.55	22.03	21.06	20.37
EDSR-LIIF [27]	**34.67**	30.96	29.00	26.75	23.71	22.17	21.18	20.48
EDSR-ours	34.60	**30.97**	**29.02**	**26.78**	**23.75**	**22.22**	**21.23**	**20.53**
RDN-LIIF [27]	**34.99**	**31.26**	29.27	26.99	23.89	22.34	21.31	20.59
RDN-ours	34.88	31.24	**29.28**	**27.01**	**23.93**	**22.38**	**21.35**	**20.63**

**Table 4:** Quantitative comparison of the ablation study (PSNR(dB)), the bold in table is the highest value.

	In-Distribution (PSNR↑)			Out-of-Distribution (PSNR↑)				
	×2	×3	×4	×6	×12	×18	×24	×30
LIIF [27]	**34.67**	30.96	29.00	26.75	23.71	22.17	21.18	20.48
LIIF + PE	34.53	30.91	28.97	26.73	23.72	22.20	21.21	20.51
LIIF + PE + FE	34.60	**30.97**	**29.02**	**26.78**	**23.75**	**22.22**	**21.23**	**20.53**
LIIF + PE + PF*	34.52	30.91	28.96	26.72	23.71	22.18	21.19	20.50

**Table 5:** Quantitative comparison of three different positional encoding approaches in Figure 3 (PSNR(dB)), the bold in table is the highest value.

	In-Distribution (PSNR↑)			Out-of-Distribution (PSNR↑)				
	×2	×3	×4	×6	×12	×18	×24	×30
LIIF [27]	**34.67**	**30.96**	**29.00**	**26.75**	23.71	22.17	21.18	20.48
LIIF + PE-hand	31.65	28.47	26.98	25.07	22.46	21.18	20.35	19.75
LIIF + PE-random	34.56	30.86	28.94	26.70	23.70	22.17	21.19	20.49
LIIF + PE-learnable	34.53	30.91	28.97	26.73	**23.72**	**22.20**	**21.21**	**20.51**

## Data Availability

No new data were created.
